# Supplementary material for: Dielectrophoresis-Based SERS Sensors for the Detection of Cancer Cells in Microfluidic Chips
Source: Biosensors (Basel). 2022 Aug 25;12(9):681. doi: 10.3390/bios12090681 (PMC9496591; doi:10.3390/bios12090681)
Supplement: Supplementary file 1 [file biosensors-12-00681-s001.zip › biosensors-1854091-supplementary.pdf]

Article

Dielectrophoresis-Based SERS Sensors for the Detection of Cancer Cells in Microfluidic Chips

Tomasz R. Szymborski [1,*], Marta Czaplicka [1], Ariadna B. Nowicka [1,2], Joanna Trzcińska-Danielewicz [3], Agnieszka Girstun [3] and Agnieszka Kamińska [1,*]

[1] Institute of Physical Chemistry, Polish Academy of Sciences, Kasprzaka 44/52, 01-224 Warsaw, Poland
[2] Faculty of Materials Engineering and Technical Physics, Poznan University of Technology, Piotrowo 3, 60-965 Poznan, Poland
[3] Department of Molecular Biology, Institute of Biochemistry, Faculty of Biology, University of Warsaw, Miecznikowa 1, 02-096 Warsaw, Poland
* Correspondence: tszymborski@ichf.edu.pl (T.S.); akaminska@ichf.edu.pl (A.K.)

Figure S1 CAM visualization of the microfluidic chip

Figure S2 COMSOL model: geometry, materials and parameters

Figure S3 COMSOL mesh of the model

Figure S4 FEM analysis of the potential and EF inside dielectrophoretic chamber

Figure S5 SERS spectra of MCF-7 for nDEP and pDEP parameters

Table S1 Parameters for the numerical calculations of the studied cancers cells using myDEP software

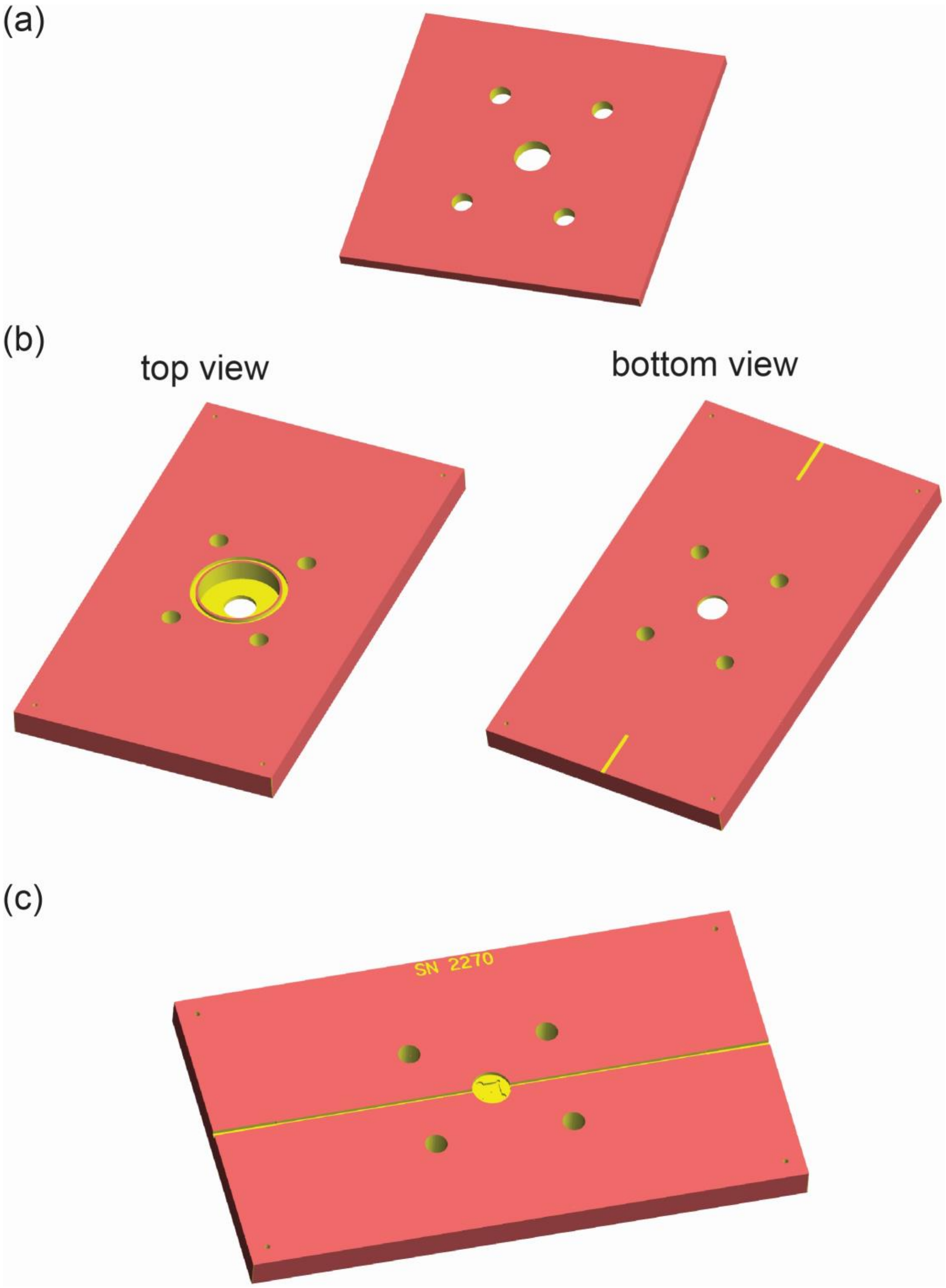

Figure S1. CAM visualization of: **(a)** the cover plate, **(b)** the top plate with microfluidic chamber, chamber for DEP electrode and sealing (top view) and two channels used for placing of the steel needles (bottom view), **(c)** the bottom plate with visible microfluidic channel and chamber for SERS-active platform.

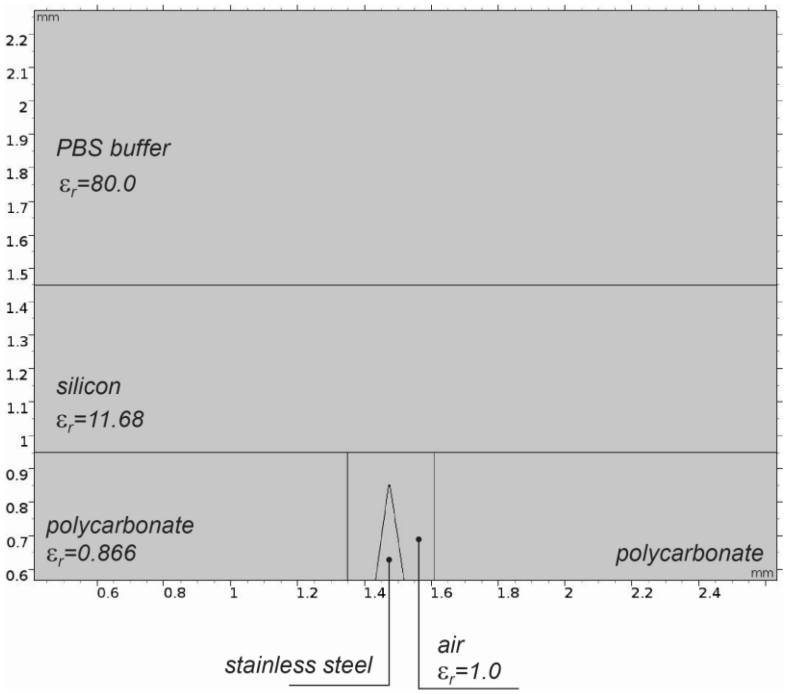

Figure S2. Geometry of the very center of the microfluidic chip with DEP electrode, SERS platform (silicon) and chamber filled with PBS buffer. The diagram indicates the materials used and the dielectric permeability coefficients (ε_r).

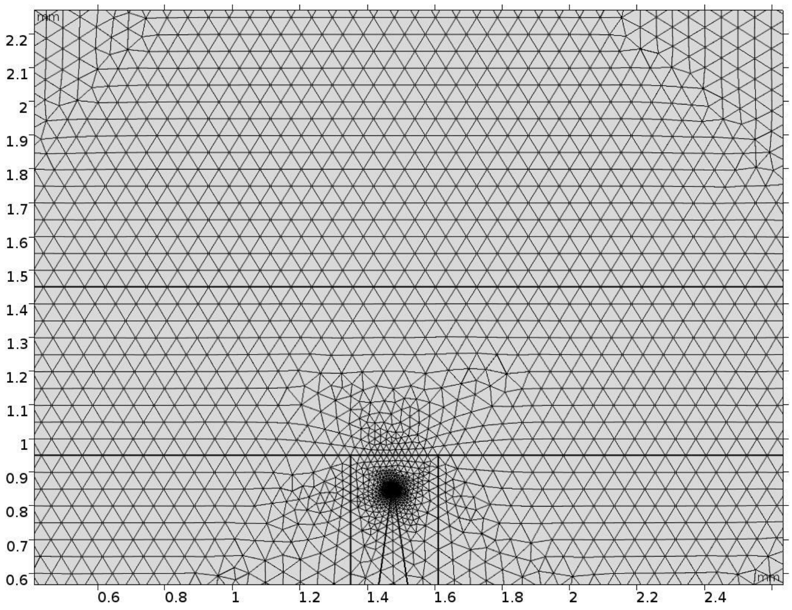

Figure S3. COMSOL model after meshing.

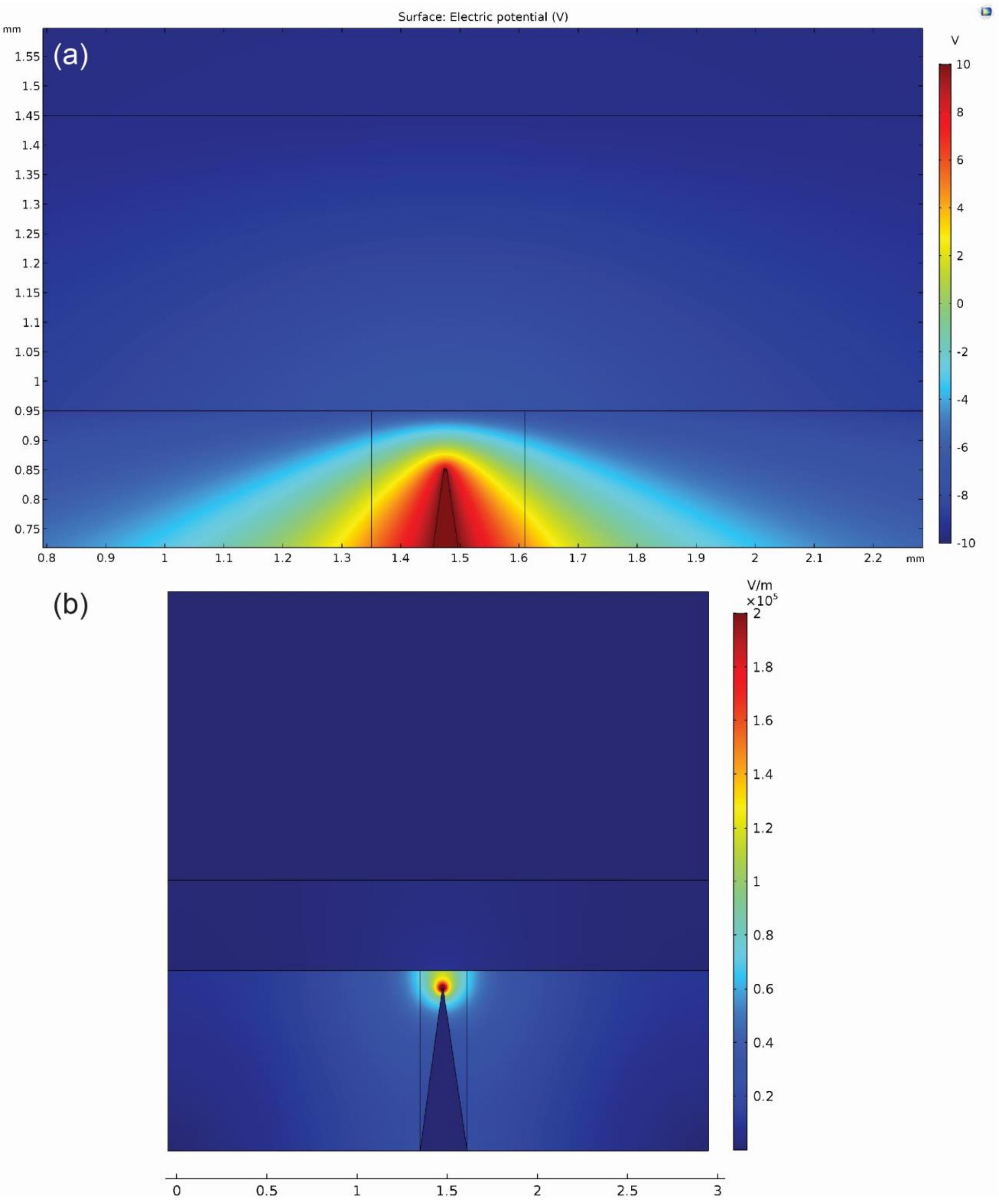

Figure S4. Visualization of the potential (U) between the top of steel electrode (+10 V) under SERS platform and top electrode (-10 V) (a) and electric field calculated using the potential from Figure S4a. Visible gradient of the electric field at the very top of the steel needle which is used as the DEP electrode.

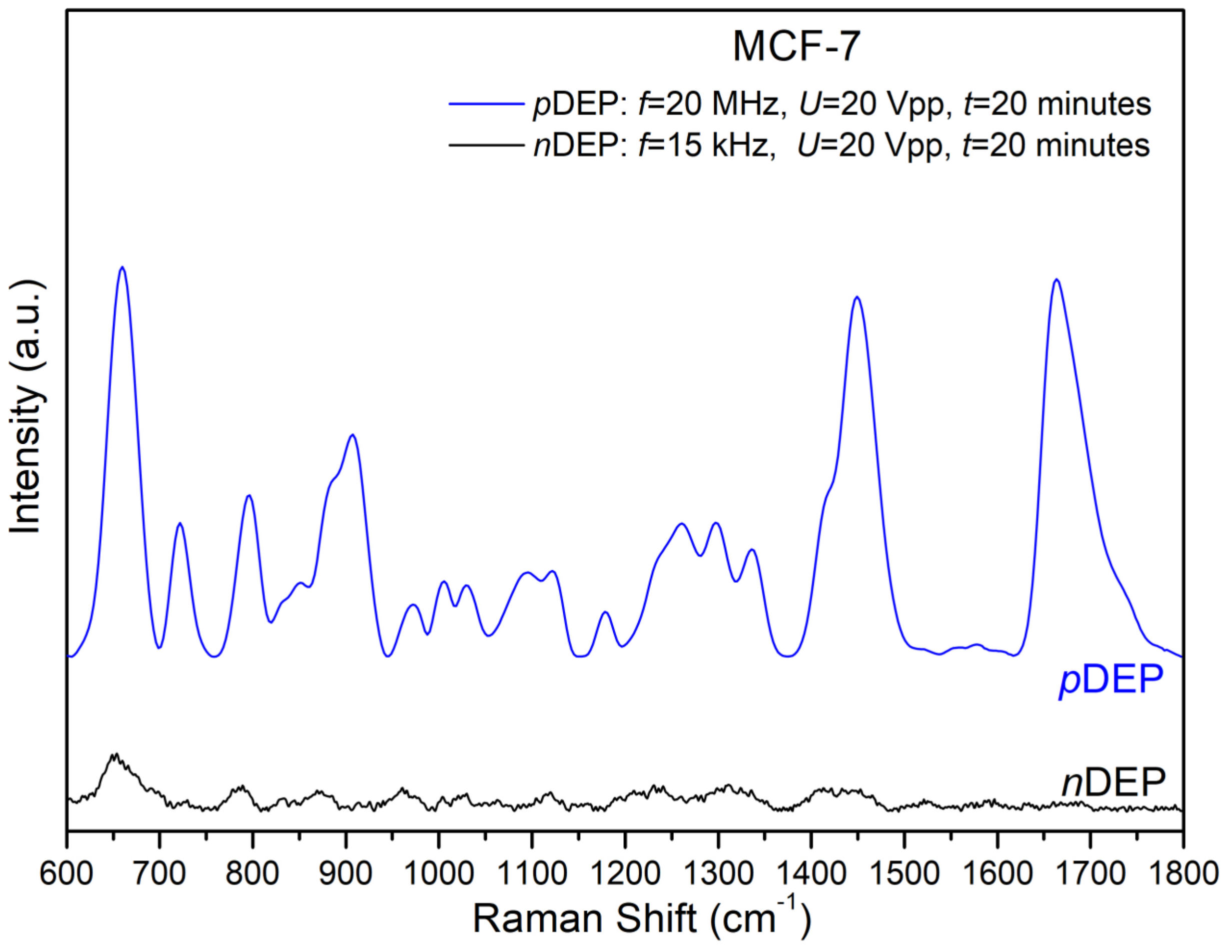

Figure S5. The selected SERS spectra of the MCF-7 cancer cells for parameters set for *n*DEP and *p*DEP.

Table S1. Parameters for the numerical calculations of the studied cancers cells using myDEP software

**Parameter**	**unit**	**MCF-7**	**MDA-MB-231**
Radius, r_{ext}	µm	9.1	6.93
**Cytoplasm**			
Electrical conductivity, σ_{cp}	S/m	1.0	0.7
Relative permitivitty, ε_{cp}	-	50.0	75.0
**Cell membrane**			
Specific membrane conductance, G_{cm}	S/m^2	0	320.0
Specific membrane capacitance, C_{cm}	mF/m^2	18.6	22.0
Reference		[73]	[74]
